# Accident or crime? About the meaning of face injuries inflicted by blunt force

**DOI:** 10.1080/20961790.2016.1229378

**Published:** 2016-12-14

**Authors:** Vera Sterzik, David Duckwitz, Michael Bohnert

**Affiliations:** a Institute of Forensic Medicine, Cantonal Hospital, St. Gallen, Switzerland; b Institute of Forensic Medicine, Julius-Maximilians-University, Würzburg, Germany

**Keywords:** Forensic science, blunt force, facial injuries, head injuries, fatalities, ear injury, retroauricular injury, accident, criminal violence

## Abstract

Injuries caused by blunt force are seen frequently in daily forensic casework. Sometimes, especially when there is less information about the surrounding circumstances, it might become difficult to figure out the cause and background of injuries: accident, criminal violence or self-infliction? In the study presented, face injuries caused by blunt force in 694 cases were analyzed comparing the injury patterns in accidents to those in crimes. It turned out injuries of the ear and retroauricular region clearly indicate a crime. Also, soft tissue injuries of nose, upper jaw, and lower jaw point towards a criminal violence, whereas tooth injuries occur with a similar frequency in both crimes and accidents.

## Introduction

In Germany, homicides and bodily harm resulting in death are most frequently caused by blunt force injuries [1,2]. An Indian study showed that people older than 60 years, for example, are five times more often victims of blunt than of sharp force [3]. Not only in crimes but also in everyday life, injuries can be caused by blunt force and occur in fall down mechanisms (falls), traffic accidents, and other accidents [4,5]. Therefore, it is a frequent challenge in forensic medicine to distinguish whether an injury was caused by an accident, self-infliction or crime [6]. The differentiation, though, is not always an easy one. The category of self-inflicted injuries is likely to show typical injury patterns (although unusual forms can occur) and is not investigated in the study presented.

In some cases, the public interest can be high and the forensic practitioner is faced with pressure as a result of people's expectations (lawyers, investigating authority and the public) [7]. It can become even more difficult to distinguish between accident and crime if there are no witnesses and the injured person is not able to make a statement, including children, people with feeble memory, and those who died.

An injury or a wound is defined as any “damage to any part of the body due to the application of mechanical force” [8]. Injuries caused by blunt force result from the application of mechanical force by a blunt object or surface and are often located in the area of head and face [3]. Injuries of this location caused by road traffic accidents have declined in the past due to seatbelts, airbags and passive pedestrian protection [9,10]. On the contrary, blunt force injuries of the head and face play an important role in fall down mechanisms, workplace and leisure activities as well as in interpersonal violence. Happenings that occur in road traffic, at workplace or while leisure activities are often easier to investigate because of their circumstances. The investigation of falls can be tricky, especially of those in domestic settings. In this context, the differentiation between injuries caused by falls and injuries caused by blow mechanisms (blows) is to be answered as well as the chronological order of several injuries [11,12]. Preuß et al. evaluated 116 cases of people falling down a stair [13]; 91% showed head injuries and 57% face injuries.

Coming back to violence, especially in blunt force violence, head and face are the location of first choice [14–16]. Ambade and Godbole evaluated 241 homicides and showed that 80% of the head injuries were caused by blunt force, whereas sharp force was only noticed in 20% [3]. Unterharnscheidt agreed that assaults are basically directed to the head and underlined that they are especially directed to the face [17]. Mützel explained that in over 50% of child maltreatments, injuries of face and oral cavity are detectable [18]. Shepherd et al. specified that in case of assault, men receive face injuries in 72%, women in 57% [15]. When children present with an injured face, several locations are indicating maltreatment rather than an accidental happening such as eyes, ears, region behind the ears, cheek and jaw region, inner side of labia oris and palatal injuries [7,11,19,20]. On the other hand, bruises on the forehead, temple, nose and chin indicate an accidental happening [19,21]. In adults, injuries of the forehead, eyebrows and tip of the nose are more likely accidental. The recognition of blunt force caused by kicks with a shoe is also important because there might be the risk of misinterpretation as blow with some kind of item, falls or traffic accidents [17]. The sole of a shoe can leave shaped marks indicating a kick [22].

To distinguish between falls and blows, the shape and number of injuries can also be of help besides the localization. Guyomarc'h et al. showed that the following criteria point towards blows with items: more than three lacerations, laceration length of 7 cm or more, comminuted or depressed skull fractures, lacerations or fractures located above the hat brim line (HBL), left-side lateralization of lacerations or fractures, more than four facial contusions or lacerations, presence of ear lacerations, presence of facial fractures, and presence of postcranial osseous and/or visceral trauma [23]. Sharkey et al. analyzed the shape, number and localization of head injuries in fall down mechanisms and blunt force recorded in autopsy cases [6]. They showed that 33% of the fall group and 70% of the blunt force group had suffered head injuries (skin lesions or skull fractures) [6]. They also pointed out that skull fractures are more likely to arise from falls and skin lesions are more likely to arise from crimes [6]. Following a study of Ström, blunt force injuries were most frequently detected on the left side of the face [24]. That is why Kremer and Sauvageau as well as Guyomarc'h et al. suggested considering the side (left or right) as well [23,25]. Analyzing the location of an injury in relation to the HBL turned out to be less helpful to distinguish between fall and blow as a single criterion [11,23,25,26].

Following the suggestion of Sharkey et al. [6] for a more complete representation of the pattern of head injuries associated with specific mechanisms, hospital admittance records were included in the study presented. To verify if it is possible to draw back conclusions about the formation of a face injury, shape, number and localization have been analyzed in 694 cases (including living and dead persons).

## Materials and methods

### Materials

All autopsies and clinical examinations (altogether 10 685 cases including 4 249 autopsies) performed by forensic experts of one Institute during a 10-year interval (2002–2012) were analyzed. Six hundred and ninety-four cases turned out to be of importance for the above-mentioned question and were included in the study presented. This number includes 622 autopsies and 72 examinations of victims alive. The cases fulfilled the following inclusion criteria:

- Blunt force impact and/or
- Injuries of head and face area due to other reasons

Furthermore, the cases did not fall within the following exclusion criteria: sharp force impact, strangulation, asphyxiation, and thermal influences (heat, cold). The data were collected and digitally stored in an anonymized way.

### Methods

The following general data have been compiled in every case:

- Age
- Sex
- Concentration of blood alcohol
- Cause of impact (given by police investigations):
  - Accident: falls (even ground, stairs, mid-height < 3 m, larger height > 3 m), workplace accident, leisure accidents and traffic accidents
  - Crime: blows with items or hand, kicks, head-butts, bites, falls because of being bumped, multiple blunt force (e.g. combination of kicks and blows)
  - Self-inflicted
- Lethal injuries and cause of death: traumatic brain injury, polytrauma, trauma to chest and/or stomach, fracture of cervical vertebrae including decapitation, embolism, brain haemorrhage, bleeding to death, others

**Figure 1.**
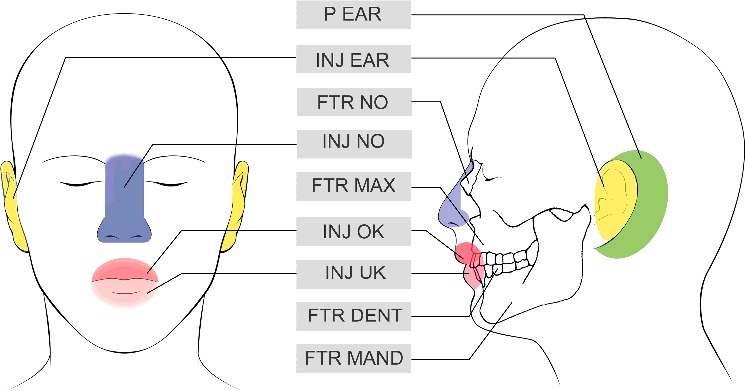
Injured areas (P EAR), injury region retroauricular (INJ EAR), injury ear region (FTR NO), fracture of nasal bone (INJ NO), injury nasal soft tissue (FTR MAX), fracture maxilla bone (INJ OK), injury soft tissue upper jaw (INJ UK), injury soft tissue lower jaw (FTR DENT), dental fracture or loss (FTR MAND) fracture mandibular bone.

Besides, the following specific data have been registered (Figure 1):

- Injury of teeth: area, tooth fracture, tooth loosening, tooth loss
- Fractures of maxilla bone: single fracture or fracture according to Le-Fort classification
- Fractures of mandibular bone
- Injury of soft tissue upper jaw
- Injury of soft tissue lower jaw
- Fractures of nasal bone
- Injury of nasal soft tissue: skin, cartilage
- Injury of the ear region (external ear and retroauricular region)

Data have been collected and stored in a Microsoft Excel 2007 database. The analysis was performed with the statistic program SPSS®. For all statistical tests, a significance level has been set at α = 0.05. Levels between 0.05 and 0.1 have been classified as slightly significant. The significance level has been calculated by using Pearson´s chi-square test and Fisher–Yates test.

## Results

### Autopsy cases (n = 622)

In the group of accidents (*n* = 588), 72.8% were men and 27.2% were women. Traffic accidents were commonest (33.1%) followed by accidental falls (30.2%). On the contrary, the group of crimes (*n* = 32) showed a balanced gender distribution of 50.0% each. The most frequent impact was multiple blunt force. The mean age of women was 57.3 years (3–97 years), whereas the mean age of men was 48.3 years (1–94 years). The most frequent cause of death was traumatic brain injury in both the accident group (42.2%) and the crime group (46.9%). The mean level of blood alcohol was 0.67‰ (highest 3.8‰) in the accident group. In the crimes group, the level of blood alcohol was higher (mean 1.21‰, highest 4.00‰).

### Forensic examinations of persons alive (n = 72)

In the group of accidents (*n* = 10), 90.0% were men and 10% were women. On the other hand, in the group of crimes (*n* = 62), 53.2% were men and 46.8% were women. Inside the group of women (*n* = 30), 96.7% were victims of a crime, and inside the group of men (*n* = 42), 78.6% were victims of a crime. The mean age of men was 36.2 years (1--94 years); the mean age of women was 32.0 years (1–85 years). The most frequent cause of injuries was multiple blunt force (35.1%), followed by blows with hand/fist (20.3%) or any item (17.6%) and falls (12.2%). The mean level of blood alcohol was 1.39‰ (highest 2.55‰) in the accident group and 1.74‰ (highest 2.00‰) in the crime group.

### Total collective (n = 694)

The total collective consisted of 70.1% men (mean age 47.3 years) and 29.9% women (mean age 53.8 years). The leading reasons for injuries were traffic accidents (69.9% male, 30.1% female), followed by accidental falls (70.1% male, 29.9% female). Criminal violence showed a nearly balanced gender distribution of 52.1% men (*n* = 49) and 47.9% women (*n* = 45).

The mean blood alcohol level of male persons was 0.80‰ (highest 4.00 ‰), whereas the mean blood alcohol level of female persons was 0.51‰ (highest 3.14‰). One person committed suicide and another one presented self-inflicted injuries (both cases were excluded from the further evaluation).

### Injuries total collective (n = 692)

**Figure 2.**
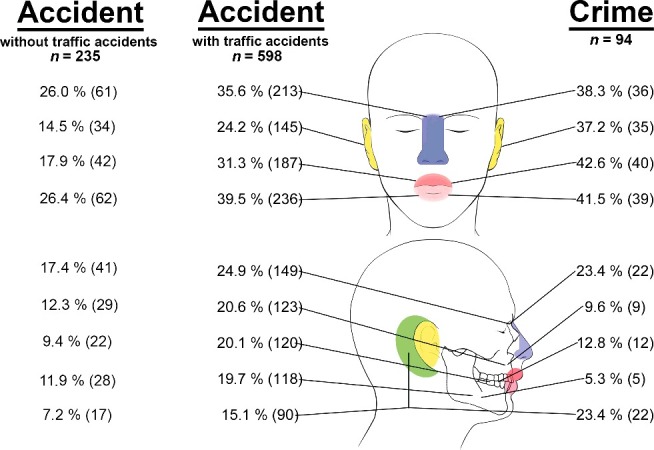
Overview on injuries in the collective crimes and accidents (with and without traffic accidents). Numbers of cases are put in brackets (). The percentage is related to the two groups “Accident” and “Crime” each for comparison of the relative frequency.

Overall, the injuries patterns of 598 accidents (including traffic accidents) were compared to 94 crime cases involving blunt force (Figure 2). Their frequency within one group (cause of impact) has been evaluated as well as their fraction within the overall injuries. Overall, 80.9% of the crime victims presented with one or more relevant head injury and so did 63.9% of the accident victims (*P* = 0.010). There were no disaccords between the trauma mechanisms given in the police reports and the ones ascertained during forensic examinations.

*Tooth injuries*: seen in 132 cases; 120 occurred due to accidents and 12 due to crimes. Within the accident group, tooth injuries were present in 20.1%. In the crime group, tooth injuries could be detected in 12.8%.
*Fractures of maxilla bone*: seen in 132 cases; 123 were caused by accidents and 9 were caused by criminal violence. Within the accident group, 20.6% showed fractures of the maxilla bone, which is significantly higher (*P* = 0.020) than in the crime group (9.6%).
*Fractures of mandibular bone*: seen in 123 cases; 118 occurred due to an accident and 5 due to a crime. In the accident group, 19.7% had a fracture of the mandibular bone. In the crime group, 5.3% presented with such an injury.
*Injuries of soft tissue intheupper jaw area*: seen in 227 cases; 187 were caused accidentally and 40 with criminal background. Within the accident group, 31.3% showed soft tissue injuries in the upper jaw area. Within the crime group, the percentage (42.6%) was significantly higher (*P* = 0.034).
*Injuries of soft tissue inthelower jaw area*: seen in 275 cases; 236 were caused by accidents and 39 were caused by criminal violence. Within the accident group, injuries of soft tissue in the lower jaw area were reported in 39.5%, and such injuries within the crime group were 41.5%.
*Injury of nasal soft tissue*: seen in 249 cases; 213 happened accidentally (*P* = 0.640) and 36 in criminal context. The overall percentage of nasal soft tissue injuries was lower in the accident group (35.6%) than in the crime group (38.3%).
*Fracture of nasal bone*: seen in 171 cases; 149 were caused by accidents and 22 by criminal events. Within the accident group, 24.9% showed such an injury, and in the crime group it was 23.4%.
*Injury of external ear region*: seen in 180 cases; 145 resulted from accidents and 35 from crimes. Injuries of the ear region were significantly (*P* = 0.008) more often seen in crimes (37.2%) than in accidental happenings (24.2%).
*Injury of retroauricular region*: seen in 112 cases; 90 were caused accidentally and 22 in criminal context. Injuries of the retroauricular region occurred significantly (*P* = 0.050) more often in crimes (23.4%) than in accidents (15.1%).

An overview of the results is shown in Figure 2 and Table 1.

**Table 1.** Overview on the percentage of injuries in the groups “Accident” and “Crime” with the relating *P*-values in the total collective.

Injury	*P*-Value	Accident (%)(*n* = 598)	Crime (%)(*n* = 94)
Teeth	0.120	20.1	12.8
Maxilla bone	0.020	20.6	9.6
Mandibular bone	0.001	19.7	5.3
Soft tissue upper jaw	0.034	31.3	42.6
Soft tissue lower jaw	0.730	39.5	41.5
Nasal soft tissue	0.640	35.6	38.3
Nasal bone	0.790	24.9	23.4
External ear	0.008	24.2	37.2
Retroauricular	0.050	15.1	23.4

The mean number of injuries per person was 2.32 in the accident group and 2.34 in the crime group (*P* = 0.940).

### Injuries of collective without traffic accidents (n = 329)

Now the traffic accidents have been excluded and a total number of 329 cases resulted including 235 accidents and 94 crimes.

*Tooth injuries*: seen in 34 cases; 22 occurred due to accidents and 12 due to crimes. Within the crime group, tooth injuries occurred relatively more frequent (12.8%, *P* = 0.420) than in the accident group (9.4%).
*Fractures of maxilla bone:* seen in 38 cases; 29 were caused by accidents and 9 were caused by criminal violence. Within the accident group, 12.3% showed fractures of the maxilla bone, and in the crime group 9.6%.
*Fractures of mandibular bone*: seen in 33 cases; 28 occurred due to an accident and 5 due to a crime. In the accident group, 11.9% had a fracture of the mandibular bone. In the crime group, 5.3% had such an injury.
*Injuries of soft tissue intheupper jaw area*: seen in 82 cases; 42 were caused accidentally and 40 with criminal background. Within the accident group, 17.9% showed soft tissue injuries in the upper jaw area. Within the crime group, the percentage (42.6%) was significantly higher (*P* = 0.000).
*Injuries of soft tissue inthelower jaw area*: seen in 101 cases; 62 were caused by accidents and 39 were caused by crimes. Within the accident group, injuries of soft tissue in the lower jaw area were reported in 26.4%, and such injuries within the crime group were 41.5%, which is significantly more frequent (*P* = 0.010).
*Injury of nasal soft tissue*: seen in 97 cases; 61 were accidental and 36 showed criminal background. The overall percentage of nasal soft tissue injuries was significantly higher in the crime group (38.3%, *P* = 0.030) than in the accident group (26.0%).
*Fracture of nasal bone*: seen in 63 cases; 41 were caused by accidents and 22 in criminal context. Within the accident group, 17.4% showed such an injury. In the crime group, it was 23.4%.
*Injury of external ear region*: seen in 69 cases; 34 resulted from accidents and 35 from crimes. Injuries of the ear region were significantly (*P* = 0.000) more often seen in crimes (37.2%) than in accidental happenings (14.5%).
*Injury of retroauricular region*: seen in 39 cases; 17 were caused accidentally and 22 in criminal context. Injuries of the retroauricular region occurred significantly (*P* = 0.000) more often in crimes (23.4%) than in accidents (7.2%).

An overview of the results is shown in Figure 2 and Table 2.

**Table 2.** Overview on the percentage of injuries in the groups “Accident” and “Crime” with the relating *P*-values in the collective without traffic accidents.

Injury	*P*-Value	Accident (%)(*n* = 235)	Crime (%)(*n* = 94)
Teeth	0.420	9.4	12.8
			
Maxilla bone	0.570	12.3	9.6
Mandibular bone	0.100	11.9	5.3
			
Soft tissue upper jaw	0.000	17.9	42.6
Soft tissue lower jaw	0.010	26.3	41.5
			
Nasal soft tissue	0.030	26.0	38.3
Nasal bone	0.270	17.4	23.4
			
External ear	0.000	14.5	37.2
Retroauricular	0.000	7.2	23.4

The mean number of injuries per person was 1.46 in the accident group and 2.34 in the crime group (*P* = 0.001).

### Injuries inflicted by fall down mechanisms (falls)

In 198 cases, injuries were caused by falls: 33.3% (*n* = 66) even grounded, 19.7% (*n* = 39) stairs, 14.1% (*n* = 28) mid-height < 3 m, and 32.8% (*n* = 65) larger height > 3 m. The mean number of injuries per person was 0.63 even grounded, 0.82 mid-height, 1.07 stairs and 2.60 larger height. Falls to even ground most frequently led to injuries of the nasal soft tissue (19.7%) and soft tissue of the lower jaw (18.2%). Falling down stairs, injuries of the external ear (28.2%) and retroauricular region (15.4%) were most common. Mid-height falls showed mainly nasal soft tissue injuries (21.4%) and injuries of the lower jaw soft tissue (17.9%). Falls from a larger height caused injuries of lower jaw soft tissue (46.2%), nasal soft tissue (40.0%), upper jaw soft tissue (36.9%), and nasal bone fractures (29.2%). The frequency of each injury within the different groups of fall down mechanisms is shown in Table 3. If an injury of the external ear was noted in the general fall group, it occurred in 37.9% because of a stair fall. Analogously, if injuries of the retroauricular region were present, the reason was a stair fall in 46.2%.

**Table 3.** Frequency of injuries within each group of fall down mechanisms.

Fall down mechanism	Even grounded (%)	Stairs (%)	Mid-height < 3 m(%)	Larger height > 3 m(%)
Teeth	1.5	–	3.6	24.6
Maxilla bone	–	2.6	3.6	27.7
Mandibular bone	–	–	–	32.3
Soft tissue upper jaw	7.6	5.1	10.7	36.9
Soft tissue lower jaw	18.2	12.8	17.9	46.2
Nasal soft tissue	19.7	10.3	21.4	40.0
Nasal bone	6.1	10.3	14.3	29.2
External ear	6.1	28.2	7.1	18.5
Retroauricular	4.5	15.4	3.6	4.6

## Discussion

Within all blunt force cases, 622 were autopsies and just 72 were examinations of people alive. This confirms Banaschak et al. who criticize that the forensic examination of people alive is not used enough although the frequency increased lately [27]. Gahr et al. showed notable differences concerning the number of forensic examinations of people alive depending on the local area [28]. In an emergency hospital at Hamburg e.g. only 2.4% of those patients with injuries due to violence were examined by a forensic practitioner as well [29]. Therefore, the number of forensic examinations performed on people alive does not at all reflect the number of people who had suffered injuries caused by violence. There might be a high estimated number of undetected cases [30].

Of all autopsy cases in our study, only 14.6% could be associated with blunt force. However, other authors reported remarkably higher percentages of 33.1% [31], 41.1% [3], and 45.0% [32]. In consent with Schulz [31] and Jäger [32], there were significantly more men (71.5%) among the autopsy cases than women (*P* = 0.08). An explanation might be that men are likely to take a higher risk regarding both the job [33] and the road traffic [34]. Among the examined persons alive, the gender distribution was balanced.

Among the autopsy cases, the mean blood alcohol concentration in victims of crimes (1.21‰) was twice as high as in victims of accidents (0.67‰). The group of people alive showed a corresponding distribution. Gerber et al. reported the involvement of alcohol in victims of crimes to be higher as in patients with any other form of injuries [35]. There are mainly two important mechanisms in victims influenced by alcohol: first, an active one when disputes are triggered and provoked (disinhibiting effect of alcohol), and, second, a passive one when a victim is vulnerable and defenceless because of being drunk. A high blood alcohol concentration does also have an effect on the injuries resulting from fall down mechanisms. Because of the victim's reduced ability to react, falls lead to more serious injuries compared to sober persons [36].

To be able to distinguish between different courses of events leading to facial injuries, two groups have been separated in this study. In agreement with Forster [37], the higher number of injuries (five or more) was pointing towards an accident. If traffic accidents could be excluded though, the number of injuries was higher after crimes. The mean number of injuries after maltreatment was 2.40, which is significantly more (*P* = 0.006) than the number of injuries after falls (1.42). The mean number of injuries after blows with some kind of blunt weapon was even higher (2.56). This is consistent with Kremer and Sauvageau, who reported the mean number of injuries after blows with blunt weapons (4.41) to be higher as the number of injuries after falls (0.49) [25]. Also, Guyomarc'h et al. showed a significantly higher number of injuries after blows (2.5) than after accidental falls (1.1; *P* = 0.00) [23]. According to that, if victims are found with several injuries to head and face, a crime is more likely to have happened than an accident.

**Figure 3.**
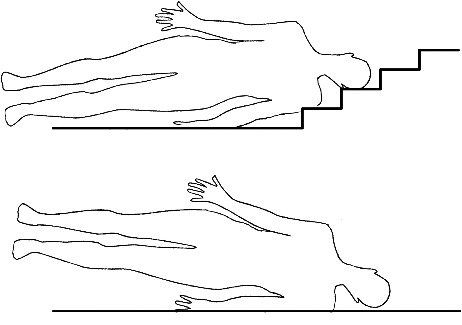
Comparison between falls on stairs and on even ground.

Injuries of the ear and retroauricular area turned out to be most important [38]. Even in the total collective, those injuries occurred significantly more often in crimes than in accidents. Despite the greatly larger group of accident cases, more injuries of the ear area were caused by criminal violence. Within this context, Guyomarc'h et al. showed that in 113 cases of head injuries, eight out of nine ear area injuries were caused by blunt force blows [23]. This may surprise on the first glimpse since the ears are located close to the HBL, which indicates injuries are caused by a fall on even ground. Taking a closer look to falls on even ground, it is apparent that the resulting injury depends on the direction of the fall. If a person is falling to his side, the shoulder will hit the floor first and the ear will be protected by the angle between shoulder and head (Figure 3). Gumpert and Maxeiner showed that the side area of the back head is dominantly hit in falls [39]. Regardless of the direction of a fall on even ground, the ears are not likely to be injured [23]. If injuries of the ear and retroauricular region occur in accidents, they are mainly caused by falls on stairs or angular structures. On the contrary to falls on even ground, the shoulder does not protect the ear region in these cases (Figure 3). Overall, injuries of the ear and retroauricular region clearly point towards a crime but need to be regarded careful if an injured person is found near a stair [40].

In both groups, the injuries of the following parts occurred most frequently: nasal soft tissue and soft tissue in the upper as well as lower jaw area. After exclusion of traffic accidents, those injuries were significantly more frequent caused by crimes. Soft tissue injuries of the upper and lower jaw were caused twice as frequently by crimes than by accidents.

In the total collective, fractures of maxilla and mandibular were most frequently caused by accidents (e.g. 93.2% of maxilla bone fractures were caused accidentally). Wagner [10] and Afaneh [41] reported similar results. When excluding traffic accidents, those injuries were noticed just slightly more often in accidents than in crimes. Therefore, relating those injuries with accidents was statistically not significant. Fractures of the nasal bone were frequent in both groups since the nose is a prominent part of the body. Due to its location, the nasal bone is also exposed to trauma since people might attentively turn their head towards an upcoming impact (in crimes as well as in other cases). There was a slight tendency that fractures of the nasal bone are more likely to be caused by crimes than by accidents, consistent with Brink [42]. Gedeon presented more significant results [43]. Analyzing 300 cases with nasal bone fracture, 61% turned out to be caused by criminal acts and just 36% were accidental [43].

The percentage of tooth injuries was pretty much the same in both the accident and crime groups. Connecting tooth injuries more likely with either crimes or accidents was not possible therefore. Included all accidents, the overall number of tooth injuries was higher resulting from a bigger case group. This fits the results of others [44,45]. The damage to dental prosthesis was not included because preliminary existing damages can make it hard to differentiate between consequence of the assault and older pre-existing damage.

## Conclusion

In contrast to “Ex ungue leonem”, the analysis of injuries does not always allow to draw back conclusions about their cause. But there are trends that can point towards one or the other direction [6], some of them with high significance. Injuries of the ear and retroauricular region are strongly associated with criminal violence and soft tissue injuries of the nose, upper and lower jaw somewhat less so. On the contrary, tooth injuries occur with a similar frequency in both crimes and accidents.

## Compliance with Ethical Standards

The authors declare that they have no conflict of interest. All procedures performed in studies involving human participants were in accordance with the relevant national legislation and local guidelines.
